# Increased Neutrophil H_2_O_2_ Production and Enhanced Pulmonary Clearance of *Klebsiella pneumoniae* in G6PD A- Mice

**DOI:** 10.21203/rs.3.rs-3931558/v1

**Published:** 2024-03-11

**Authors:** Benjamin E Zuchelkowski, Hernán F Peñaloza, Zeyu Xiong, Ling Wang, Eugenia Cifuentes-Pagano, Elizabeth Rochon, Minying Yang, Sebastien Gingras, Mark T Gladwin, Janet S Lee

**Affiliations:** University of Pittsburgh School of Medicine; University of Pittsburgh School of Medicine; Washington University in St. Louis; University of Iowa; Pittsburgh Heart, Lung, Blood and Vascular Medicine Institute; University of Maryland, University of Maryland; Pittsburgh Heart, Lung, Blood and Vascular Medicine Institute; University of Pittsburgh School of Medicine; University of Maryland, University of Maryland; Washington University School of Medicine

## Abstract

The X-linked A^−^ variant (rs1050828, Val68Met) in *G6PDX* accounts for glucose-6-phosphate (G6PD) deficiency in approximately 11% of African American males. This common, hypomorphic variant may impact pulmonary host defense and phagocyte function during pneumonia by altering levels of reactive oxygen species produced by host leukocytes. We used CRISPR-Cas9 technology to generate novel mouse strain with “humanized” G6PD A- variant containing non-synonymous Val68Met single nucleotide polymorphism. Male hemizygous or littermate wild-type (WT) controls were inoculated intratracheally with *K. pneumoniae* (KP2 serotype, ATCC 43816 strain,10^3^ CFU inoculum). We examined leukocyte recruitment, organ bacterial burden, bone marrow neutrophil and macrophage (BMDM) phagocytic capacity, and hydrogen peroxide (H_2_O_2_) production. Unexpectedly, G6PD-deficient mice showed decreased lung bacterial burden (p=0.05) compared to controls 24-h post-infection. Extrapulmonary dissemination and bacteremia were significantly reduced in G6PD-deficient mice 48-h post-infection. Bronchoalveolar lavage fluid (BALF) IL-10 levels were elevated in G6PD-deficient mice (p=0.03) compared to controls at 24-h but were lower at 48-h (p=0.03). G6PD A- BMDMs show mildly decreased *in vitro* phagocytosis of pHrodo-labeled KP2 (p=0.03). Baseline, but not stimulated, H_2_O_2_ production by G6PD A- neutrophils was greater compared to WT neutrophils. G6PD A- variant demonstrate higher basal neutrophil H_2_O_2_ production and are protected against acute *Klebsiella* intrapulmonary infection.

## Background

Glucose-6-phosphate dehydrogenase (G6PD) deficiency is among the most common red blood cell (RBC) enzymopathies worldwide^1^. It affects over 500 million individuals^1^. While it is predominantly clinically silent, complete loss of enzyme activity is lethal in human and mouse models^1^. G6PD deficiency results in decreased production of NADPH via the pentose phosphate pathway, leading to impaired antioxidant activity by key NADPH-dependent pathways, impairing glutathione reductase^1^, and increasing oxidized glutathione and hydrogen peroxide (H_2_O_2_) levels. Over 200 causative mutations in the *G6PDX* gene are associated with known clinical variants that are classified into five categories based on residual enzyme activity ^2,3^. The A-, or African, variant is a class III variant, indicating it causes “mild” disease ^4^. It is the most common G6PD variant and affects up to 11% of African American men ^5^.

Despite being one of the most widely-studied enzyme deficiencies, key questions remain regarding the functional relevance of G6PD deficiency due to seemingly conflicting findings. A meta-analysis previously reported that G6PD deficiency confers protection against malaria potentially due to enhanced early phagocytosis of infected erythrocytes ^6,7^. This protection is thought to have exerted selective pressure in malaria-endemic regions, thus accounting for its global prevalence.

This is in contrast to recent investigations suggesting that G6PD deficiency may impair key immune functions, including inflammasome activation ^8^, reduced NETosis ^9^, and impaired macrophage and neutrophil oxidative burst ^10^. Trauma patients with G6PD deficiency have longer duration of antibiotics and hospital stays, and circulating monocytes from these patients demonstrate higher levels of oxidative stress ^11^. Furthermore, severe G6PD deficiency defined as < 5% enzyme activity, can phenocopy chronic granulomatous disease (CGD), which is due to an inherited mutation in one of five NADPH oxidase genes leading to severely decreased ROS production, and presents with a predisposition to recurrent catalase-positive bacterial and fungal infections ^9^. In G6PD deficient patients, increased susceptibility to infections have been reported and include, but are not limited to, pneumonia without microbiologic diagnosis ^12^, and specific *pathogens Serratia marcescens, Chromobacterium violaceum, Aspergillus* spp. causing disseminated aspergillosis ^13^, and bronchial infections in G6PD A- trauma patients ^11^.

The role of G6PD activity in the immune response to infection remains controversial, with a variety of observtions noted in different experimental models, with a variety of methods used to inhibit enzyme activity ^14,10^, which may impact generalization of results across studies. For example, one report observed impaired inflammasome activation in human G6PD-deficient peripheral blood monocytes (PBMCs) and in siRNA-mediated *G6PD* knockdown in LPS-primed THP1 cells ^8^. Another demonstrated no difference in survival between G6PD-deficient mice and littermate controls after cecal ligation and puncture-induced sepsis ^14^, while still another used a small-molecule inhibitor of G6PD to show that G6PD inhibition suppresses neutrophil oxidative burst ^10^.

Individuals with severe G6PD deficiency may be predisposed to recurrent bacterial lung infections, suggesting that residual G6PD activity may play a role in determining host leukoctye function. Moreover, the role of reactive oxygen species (ROS) has not been well-understood in the context of G6PD A- leukocytes given that G6PD catalyzes the generation of NADPH from NADP^+^ and curtails hydroxyl free radical conversion from H_2_O_2_. While there are multiple cellular sources of NADPH in nucleated cells, evidence suggests they may be insufficient to maintain an adequate pool of NADPH ^15^. Therefore, inhibition of G6PD may alter a cell’s ability to handle its oxidative load; consistent with this idea, one study suggests that total ROS levels are increased in G6PD deficient monocytes from trauma patients ^11^. Furthermore, hypochlorous acid (HOCl) production by neutrophil myeloperoxidase (MPO), a vigorous antimicrobial killing compound^16,17^, uses H_2_O_2_ as its substrate. It is therefore possible that leukocytes with deficient G6PD activity may be able to harness the excess H_2_O_2_ for enhanced antimicrobial activity.

To our knowledge, no study has examined the direct effect of the hypomorphic G6PD A- variant in a mouse model of bacterial acute lung infection. Closer examination of this variant may have important clinical implications since approximately 11% of African American males are hemizygous for this X-linked variant. We hypothesized that G6PD deficiency due to the A- variant alters pulmonary host defense, neutrophil and macrophage function, by altering ROS levels produced by host leukocytes. To address this question we used a novel humanized murine model of G6PD A- to examine the host response to *K. pneumoniae* acute lung infection, a model where early neutrophil antimicrobial function is essential for host defense ^18^. Our study demonstrates that decreased G6PD activity is protective against *K. pneumoniae* infection due to altered neutrophilic ROS production.

## Methods

### Animals

We used CRISPR-Cas9 technology ^19,20^ to create a humanized murine model of G6PD deficiency containing the G6PD A- variant (rs1050828, Val68Met). Valine 68 was substituted to Methionine using an oligouncleotide template for homology-directed repair (HDR) containing the homologous human DNA sequence. Founder mice and N1 offspring were confirmed through sequence analysis and a stable colony was obtained through strategic back-crossing to wild-type mice. We previously reported that erythrocyte G6PD activity from hemizygous male mice was approximately 5% of WT-G6PD activity^21^.

For *in vivo* pneumonia studies, a sample size of 8 animals per group was determined *a priori* to achieve a power of 0.83 for a 2-tailed test using an α of 0.05. A skilled technician who was blinded to the biological hypothesis performed the inoculations, monitoring, and harvest. Only male mice were used in this study as G6PD deficiency is X-linked. Male mice aged 12 – 14 weeks were used for experiments with WT littermates used as controls. Briefly, mice were inoculated via direct intratracheal instillation with *K. pneumoniae* at 10^3^ CFU inoculum. Animals were sacrificed and tissues were harvested after 24- and 48-hours in independent experiments.

### Euthanasia and Ethical Approval

Animals were euthanized at time of experimental procedures using overdose isoflurane followed by exsanguination via inferior vena cava. The mice were monitored carefully and euthanized if they met predefined criteria for euthanasia. All procedures were performed with approval of the Institutional Animal Care and Use Committee at the University of Pittsburgh (protocol numbers 18052522 and 18063096). All procedures are in accordance with relevant guidelines and regulations including ARRIVE guidelines.

### *Klebsiella pneumoniae* strains

*K. pneumoniae* strain: The strain used in this study was *K. pneumoniae* strain 43816 serotype 2 (American Type Culture Collection, Manassas, VA).

### *K. pneumoniae* inoculation

*K. pneumoniae* strains were grown overnight in tryptic soy broth (TSB) for 18 hours shaking at 250 rpm at 37°C. A 1:100 dilution of bacteria in TSB was then incubated at 37°C for 1.5 hours. The bacterial inoculum was prepared in PBS to OD_600nm_ = 0.2. Mice were instilled with 10^3^ CFU inoculum by the intratracheal route. We have previously described a detailed method of intratracheal administration of bacteria by direct visualization ^22,23^.

### Bronchoalveolar Lavage

A detailed method of bronchoalveolar lavage fluid (BALF) collection has been previously described ^24,25^. Briefly, at the specified times, mice were anesthetized with isofluorane. The thoracic cavity was opened by midline incision. The trachea was exposed and cannulated with a 20-gauge catheter, which was secured with a 2–0 silk suture. The left main stem bronchus was identified and divided at the hilum, and the entire left lung was removed. BAL was performed on the right lung with PBS containing 0.6 mM EDTA instilled in one aliquot of 0.6 mL, followed by three aliquots of 0.5 mL.

### Determination of bacterial burden

The left lung, spleen, and right medial lobe of the liver were removed, and placed in 1 ml of sterile water. Tissues were immediately homogenized on ice with a tissue homogenizer. Blood was drawn in citrate-phosphate-dextrose treated 23-gauge syringes from the inferior vena cava of each anesthetized mouse. Serial 1:10 dilutions of isolated whole blood and tissue homogenates in sterile PBS were plated on tryptic soy agar. Plates were incubated for 18 h at 37°C after which colonies were counted.

### Isolation and culture of bone marrow-derived macrophages (BMDMs)

Isolation and differentiation of bone marrow-derived myeloid cells to non-activated macrophages has been previously described ^26^. Briefly, bone marrow cells from WT and G6PD A- mice were allowed to adhere and cultured in differentiation media (DMEM, 20% neonatal calf serum, 30% L929 conditioned medium, 1% penicillin-streptomycin) at a density of 1 × 10^6^ cells/ml in 100 × 20mm sterile petri dishes for 7 days. Medium was replaced at day 5. After 7 days, differentiated BMDMs were cultured in 24-well plates at a density of 2.5 × 10^5^ cells/ well in a final volume of 500 μL 24-hours prior to subsequent use in *in vitro* pHrodo phagocytosis assay.

### Isolation of bone marrow neutrophils

Extraction of bone marrow cells was performed as described previously ^27^, and neutrophils were isolated from cell suspensions via negative selection (Miltenyi Neutrophil Isolation Kit, #130–097-658) according to the manufacturer’s instructions. Isolated cells were diluted in serum-free OptiMEM to a final concentration of 4×10^4^ cells per 30 μL media. The final cell suspension was >95% polymorphonuclear cells as determined by cytospin preparations. These cells were immediately used in the modified Amplex Red assay as described below. Unused cell suspensions were centrifuged at 300 *× g* for 10 minutes, lysed, and pellets stored at −80°C.

### pHrodo^™^ *in vitro* Phagocytosis Assay

*K. pneumoniae* strain 43816 serotype 2 was grown to OD_600nm_ = 0.2 then heat-killed at 60°C for one hour. Heat-killed bacteria were centrifuged at 12,000 rpm for 2 min, resuspended in 100 mM sodium bicarbonate, centrifuged at 12,000 rpm for 2 min, then resuspended in 200 μL sodium bicarbonate. pHrodo^™^ iFL Green STP Ester dye (ThermoFisher #P36012) at 10 mg/mL was diluted in the bacterial suspension to 1 mM, then incubated for one hour in the dark. Unconjugated dye was removed through successive washing steps with PBS and 100% methanol. BMDMs at a density of 2.5×10^5^ cells/well were infected with heat-killed, labeled-bacteria at MOI=10 for 90 min. Plates were centrifuged at 1500 rpm for 5 min at 4°C to synchronize phagocytosis. As a negative control, cytochalasin D [10 μM] was applied to selected wells one hour prior to infection to inhibit phagocytic uptake. Adherent cells were isolated by vigorous washing with ice cold PBS. pHrodo^™^-positive macrophages were examined by flow cytometry analysis. The phagocytosis of pHrodo labeled KP2 in BMDMs was detected as green fluorescence positive events by BD FACScalibur. Four wells of WT and four wells of G6PD A- BMDMs were obtained from one mouse each as technical duplicates.

### *In vivo* Phagocytosis Assays

For measuring *in vivo* phagocytosis, pHrodo labeled *K. pneumoniae* was instilled intratracheally into mice at a concentration of ~5 × 10^7^ CFU in 0.1 mL total volume. After one hour, mice were euthanized and BAL was performed using 1-mL of 0.6mM EDTA in PBS followed by three sequential lavages of 0.9-mL. BAL cells were labeled with fluorochrome-conjugated antibodies against Ly6G PE and F4/80 APC. The flow cytometry data were acquired on BD FACSCalibur. The positive events were back-calculated as absolute BAL cell counts based upon total cell counts.

### Enzyme-Linked Immunosorbent Assays (ELISAs)

ELISA Quantikine-sets for murine interleukin-1β (IL-1β), interleukin-10 (IL-10), tumor necrosis factor-alpha (TNF-α), and granulocyte-macrophage colony-stimulating factor (GM-CSF) were purchased from R&D Systems and the levels of cytokines in BALF were measured according to the manufacturer’s instructions (Minneapolis, Minnesota).

### Measurement of Extracellular H_2_O_2_ Production

The measurement of extracellular H_2_O_2_ production was performed using BMDMs and bone marrow neutrophils using a modified Amplex red assay. Briefly, H_2_O_2_ production was quantified in whole cells by Amplex^®^ Red as previously described^28–30^. Either bone marrow derived macrophages or bone marrow derived neutrophils were prepared as described above, resuspended in OptiMEM and then plated (4 × 10^4^ cells/well) into 96-well plate in assay buffer (25 mM Hepes, pH 7.4, containing 0.12 M NaCl, 3 mM KCl, 1 mM MgCl_2_) supplemented with 0.1 mM Amplex^®^ Red, and 0.32 U/ml of horse radish peroxidase (HRP). The reaction was started by addition of 5μM phorbol 12-myristate 13-acetate (PMA). Fluorescence measurements were made using a Biotek Synergy 4 Hybrid Multi-Mode Microplate Reader with a 530/25 excitation and a 590/35 emission filter. The reaction was monitored at 37 °C for 1 h. A standard curve of known H_2_O2 concentrations was included on each plate. NADPH oxidase (NOX) activity was obtained by calculating the rate of H_2_O_2_ production as RFU/min/40,000 cells after subtracting the equivalent value given by cells treated with 300 U/ml of catalase. Data are expressed as fold change of WT vehicle control.

### Phagocytosis Marker Assay

BMDMs were stimulated with LPS at 100 ng/mL (ULTRA PURE LPS from *Escherichia coli* O111:B4, #421, List Labs) or saline vehicle for 4 hours. Flow cytometry analysis was performed using antibodies against the following markers: CD16 (BioLegend, #158010), CD18 (BioLegend, #101405), CD32 (BioLegend, #156406), CD21/35 (BioLegend, #123418), CD36 (BD Biosciences, #562477), CD64 (BD Biosciences, #740622), CD204 (BD Biosciences, #748091), CD206 (BioLegend, #141719), CD11c (BioLegend, #117348), CD11b (BD BioSciences #553311), MARCO (R&D, #FAB2956N).

### Statistical Analysis

A Student *t* test was used to compare differences between two groups. A 2-tailed Mann-Whitney U test was used for data that was not normally distributed. Differences were considered significant for p-values < 0.05, and significance was Bonferroni-adjusted for multiple comparisons where appropriate. All statistical analysis was performed using GraphPad Prism 9.5.0.

## Results

### G6PD A- mice show lower lung bacterial burden and decreased extrapulmonary dissemination following Klebsiella pneumoniae intrapulmonary infection

**Figure 1:**
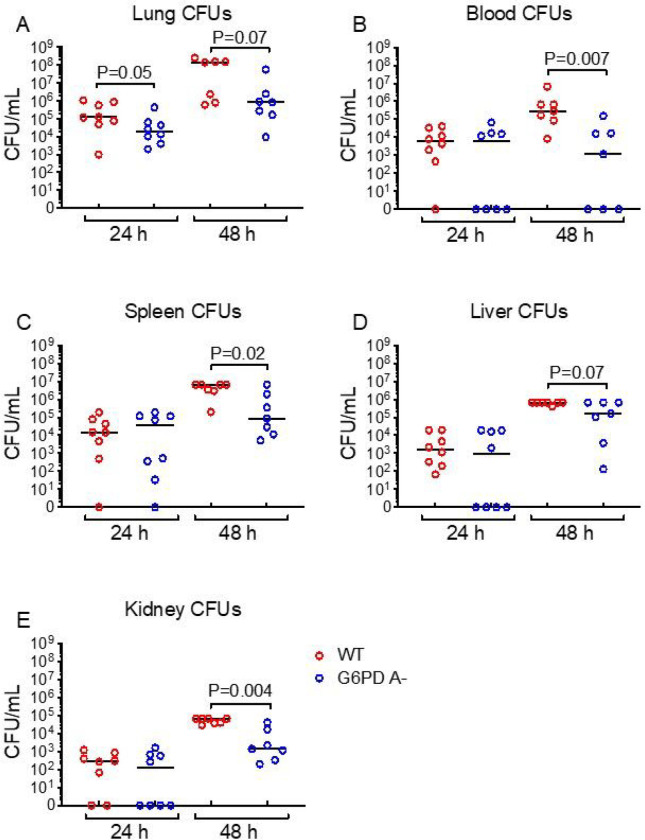
G6PD A- mice show reduced intrapulmonary bacterial burden and extrapulmonary dissemination during *Klebsiella pneumoniae* pneumonia. Mice underwent intratracheal inoculation with *K. pneumoniae* (10^3^ CFU inoculum). Organs and blood were harvested at the times indicated, and total CFUs were quantified. **A**, lung tissue CFU/mL tissue homogenate; **B**, blood CFU/mL tissue homogenate; **C**, spleen tissue CFU/mL tissue homogenate; **D**, liver CFU/mL tissue homogenate; **E**, kidney CFU/mL tissue homogenate. Data were analyzed by Mann-Whitney U test, bar = median. Red open circles indicate WT mice, blue open circles indicate G6PD A- mice. Each point indicates individual mice.

We tested the hypothesis that G6PD A- alters the clearance and dissemination of *K. pneumoniae* from the lungs following acute intrapulmonary infection. At 24 h post-infection, G6PD A- mice showed significantly lower lung bacterial burden compared to WT littermates and a trend toward lower lung burden after 48 h (Fig. 1A). At 48 h post-infection, G6PD A- mice showed significantly less bacteremia (Fig. 1B) and reduced organ dissemination to spleen, liver, and kidney (Fig. 1C–E).

**Figure 2:**
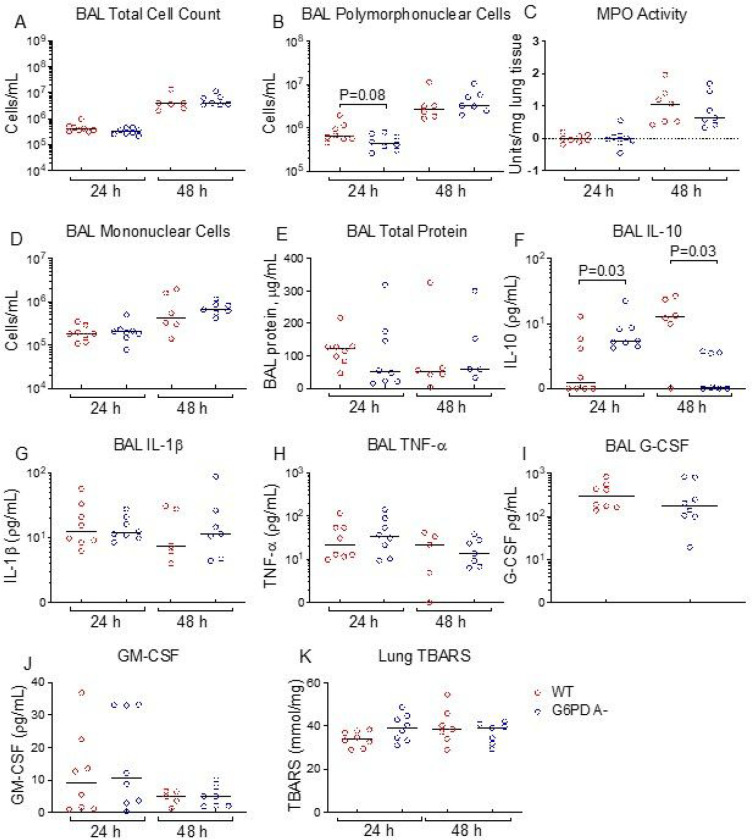
G6PD A- show increased IL-10 concentrations early during *Klebsiella pneumoniae* pneumonia. Mice underwent intratracheal inoculation with *K. pneumoniae* (10^3^ CFU inoculum). Organ tissue homogenates and blood were harvested at the times indicated, and total CFUs were quantified. **A**, total cell count in BAL, cells/mL; **B**, polymorphonuclear cells/mL BAL; **C**, myeloperoxidase (MPO) activity U/mg lung tissue; **D**, mononuclear cells/mL BAL; **E**, total protein μg/mL BAL; **F-J**, cytokines and chemokine concentrations in BAL; **K**, TBARS in lung tissue homogenate mmol/mg. Data were analyzed by Mann-Whitney U test, bar = median. Red open circles indicate WT mice, blue open circles indicate G6PD A- mice. Each point indicates individual mice.

Given the enhanced host defense observed in the G6PD A- mice, we examined the inflammatory cell recruitment profile of BALF at 24 and 48 h. Compared to WT mice, G6PD A- mice showed similar total BALF cell counts, neutrophil, and mononuclear cell numbers/mL (Fig. 2A, B, D). Lung tissue myeloperoxidase levels, a marker of total neutrophil content in lung tissue homogenates, were similar between G6PD A- and WT mice (Fig. 2C). Total BALF protein, a marker of lung microvascular permeability, was not different between G6PD A- and WT mice (Fig. 2E). These results suggest that the G6PD A- variant is associated with enhanced early host immune response and reduced extra-pulmonary bacterial dissemination.

### G6PD A- mice show similar inflammatory cytokine profile in the lung as WT mice during infection

Previous studies demonstrate that G6PD deficient monocytes from human subjects exhibit increased *ex vivo* LPS-induced IL-10 levels, and greater *in vivo* IL-1β, IL-6, and IL-10 levels in the serum and peritoneal lavage fluid from LPS-treated G6PD-deficient mice ^14,31^. Consistent with these prior human studies, G6PD A- mice had significantly higher levels of IL-10 in the BALF compared to WT mice (Fig. 2F) after 24 h. However, after 48 h, BALF IL-10 levels in G6PD A- mice were lower than those measured from WT mice. No differences were observed in the master cytokines IL-1β, TNF-α, or GM-CSF (Fig. 2F–J). Further, measurements of lung tissue malondialdehyde, a major lipid oxidation product, using the thiobarbituric acid reactive substance (TBARS) assay found similar levels in G6PD A- and WT mice (Fig. 2K).

### Bacterial phagocytosis is not altered in G6PD A- bone-marrow derived macrophages in vitro, and alveolar macrophages or neutrophils in vivo

**Figure 3:**
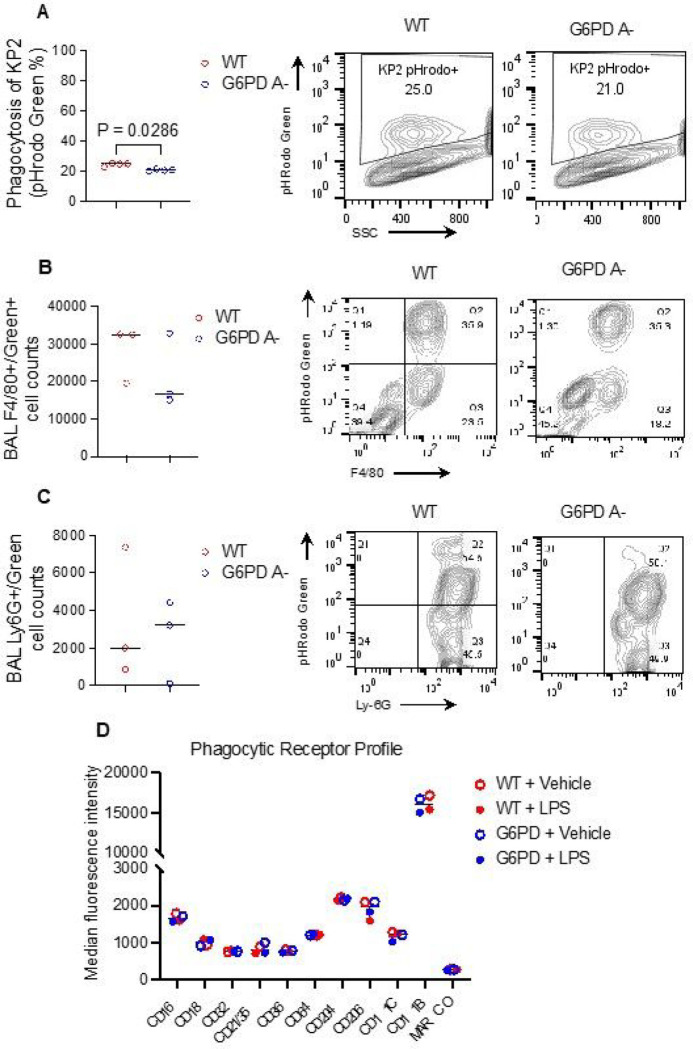
*In vitro* phagocytosis of KP2 by G6PD A- bone marrow derived macrophages (BMDMs) and *in vivo* phagocytosis by airspace macrophages. **A,** pHrodo-labeled KP2 phagocytosis by G6PD A- and WT bone marrow-derived macrophages. **B,C**pHrodo-labeled KP2 phagocytosis by G6PD A- and WT alveolar macrophages **(B)**and neutrophils **(C)**. **D,** Profile of classic phagocytic markers on bone marrow-derived macrophages was assessed using flow cytometry. Macrophages were exposed to vehicle (PBS) or 100 ng/mL LPS for 4 hours prior to measurement. For **A-C,** data points represent technical replicates in a single representative experiment, differences by Mann-Whitney U test. For **D,** each point represents at least three pooled technical replicates from one representative experiment, two-way ANOVA with Tukey multiple comparisons. For **A-D,** bars = median.

We tested whether multiple cell types, including BMDMs, alveolar macrophages and bone marrow neutrophils from G6PD A- mice exhibit increased phagocytosis of *K. pnuemoniae*. To visualize labeled bacteria entering newly formed phagolysosomes, BMDMs were isolated, differentiated and pooled from G6PD A- and WT littermate mice and exposed to pHrodo-labeled *K. pnuemoniae* (MOI = 10). We observed slightly decreased phagocytosis in the G6PD A- macrophages compared to WT BMDMs (Fig. 3A). In the lung, there were no genotype differences in pHrodo signal intensity in airspace macrophages (Fig. 3B) or neutrophils (Fig. 3C). Overall, phagocytosis rate was 32–45%, and there were few Ly6G-positive cells, and ~ 82% F4/80-positive macropahges. We also compared the phagocytic receptor profile expressed by bone marrow-derived macrophages from G6PD A- and WT mice. We did not observe any differences between the receptors surveyed involved in bacterial uptake (Fig. 3D). These experiments suggest G6PD A- does not impact bacterial phagocytosis and phagocytic receptor expression.

### Baseline neutrophil H_2_O_2_ production is increased in G6PD A- compared to WT

**Figure 4:**
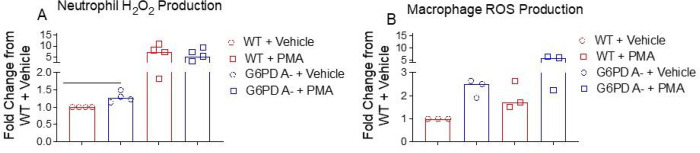
Baseline H_2_O_2_ levels are increased in unstimulated G6PD A- neutrophils but not in macrophages. The rate of H_2_O_2_ production in response to phorbol 12-myristate 13-acetate (PMA) was assessed using a modified Amplex Red assay in bone marrow neutrophils (A) and bone marrow derived macrophages (B). In (A), three independent experiments were performed with each point representing a separate biologic replicate. In (B), two indepdent experiments were performed with each point representing a separate biologic replicate. Gray shade represents samples stimulated by PMA. Data were analyzed by Mann-Whitney U test, bar = median, * p < 0.05

Mononuclear phagocytes and polymorphonuclear cells are critically important for host control of *K. pneumoniae* infection^32,33^. We performed a modified Amplex Red assay to compare the rate of H_2_O_2_ production between WT and G6PD A- neutrophils and macrophages. We observed significanly higher baseline H_2_O_2_ production in the G6PD A- neutrophils (Fig. 4A) but not macrophages (Fig. 4B) compared to WT. In each cell type, the H_2_O_2_ production was not different following PMA stimulation, although the total H_2_O_2_ levels trended higher in G6PD A- neutrophils and macrophages. The G6PD A- BMDM NADPH production was ~ 20% reduced compared to WT G6PD activity (Supplementary Fig. 1). These results suggest that decreased G6PD activity influences H_2_O_2_ buffering capacity in neutrophils while macrophages are able to compensate and maintain WT levels of H_2_O_2_ even in the absence of a fully functional G6PD enzyme.

## Conclusions

In this study, we developed a humanized model of G6PD A- to explore responses to pneumonia, using a common pathogenic strain of *K. pneumoniae*. We found that G6PD A- mice exhibit a favorable pulmonary host defense during *K. pneumoniae* infection as demonstrated by reduced lung bacterial burden and extrapulmonary dissemination compared to WT littermates. Despite enhanced pulmonary host defense, the G6PD A- mice showed similar airspace neutrophil counts, inflammatory cytokine profile, and phagocytosis as WT mice. We observed higher baseline H_2_O_2_ production from G6PD A- neutrophils but not macrophages compared to WT littermates. Together, our data suggest that the enhanced immune response may be mediated in part by differences in baseline neutrophilic H_2_O_2_ production.

This study suggests a complex relationship between G6PD deficiency and the role of ROS production in innate immunity. The oxidative burst in neutrophils is a principal component of the innate immune defense against bacterial and fungal pathogens, and neutrophils are the main drivers of the innate response during bacterial infection ^18^, especially with regard to ROS production by NADPH oxidase ^34^. Treatment with a small-molecule inhibitor of G6PD lowered *in vitro* oxidative phosphorylation in mouse and human neutrophils, while the NADP+/NADPH ratio was less affected in mouse macrophages ^10^. Human G6PD A- circulating monocytes have higher total levels of *in vitro* PMA-induced ROS compared to non-G6PD-deficient monocytes, while neutrophils from the same subjects did not exhibit differences in PMA-induced ROS ^11^. Increased total ROS production in G6PD A- is likely three-fold: 1) due to inadequate NADPH substrate necessary for glutathione recycling and neutralization of ROS; 2) increased MPO-mediated HOCl production from H_2_O_2_, and 3) increased activity of NOX isoforms ^35^. Interestingly, we did not observe genotype differences in lung tissue homogenate TBARS as a marker of oxidative stress during the course of *K. pneumoniae* infection (Fig. 2K). Inability to detect a difference in tissue may be due to the lower sensitivity of this method to detect cellular oxidative stress in a specific cell type. G6PD deficiency due to the A- variant is typically clinically silent unless these patients are exposed to overwhelming oxidative stress ^1^. While it is not known exactly how phagocyte H_2_O_2_ production exerts its anti-microbial properties ^36^, the current study raises the possibility that G6PD A-exerts its enhanced response to *K. pneumoniae* at least in part via differential H_2_O_2_ production. We further speculate that conversion of H_2_O_2_ to HOCl may account for a component of increased bacterial killing by G6PD A- leukocytes.

In the current study, we observed increased bronchoalveolar IL-10 levels in the G6PD A- mice versus WT in the initial 24 h period, followed by lower levels in G6PD A- mice at 48 h. IL-10 has been shown to enhance phagocytosis in monocytes and macrophages ^37^. In bacterial infections such as *S. pneumoniae*, IL-10 is important for clearing extracellular bacteria despite having sustained elevation of inflammatory markers and neutrophil infiltration in the lung ^38^. Still others have shown during *K. pneumoniae* (ATCC 43816) infection, the absence of IL-10 may lead to more robust bacterial clearance ^39^, although this phenotype may be strain specific as a second study has shown that during high doses of carbapenem-resistant *K. pneumoniae*, IL-10 deficiency leads to increased mortality and uncontrolled lung inflammation^40^. A previous *in vitro* study found that *IL-10* transcript and protein levels were markedly increased in G6PD deficient macrophages compared to WT and, depending on the activation status of the macrophage with LPS, PMA, or LPS + PMA, shifted the G6PD macrophages to an anti-inflammatory cytokine profile, with associated increases in NF-κB, Sp3, CREB, and PKCδ signaling factors ^31^. Further studies are needed to determine the exact role of G6PD in the regulation of IL-10 production *in vivo*.

Based on the enhanced clearance of *K. pneumoniae* in the circulation and extra-pulmonary organs observed in our humanized mouse, this raises the possibility of parallels to the immune-protective effect of G6PD A- against malaria. The proposed protective mechanism against malaria rests on the premise of enhanced erythrophagocytosis of infected RBCs. We did not observe differences in neutrophil or macrophage phagocytosis, or in classic phagocytic surface marker profiles. However, more recent evidence from G6PD-deficient mice infected with *Plasmodium berghei* demonstrate a survival advantage, less severe experimental cerebral malaria, and milder acute liver injury via an attenuated proinflammatory response compared to WT controls^41^. In that study, serum IL-10 levels were not different between G6PD deficient and WT mice, although proinflammatory cytokine levels were lower in G6PD deficient mice^41^.

We utilized a humanized murine model of the G6PD A- variant and examination of phagocytosis and H_2_O_2_ production across multiple cell types. Prior studies have relied upon *in vitro* chemical inhibition of G6PD or mutations outside the causative V68M SNP. Those methods may fail to capture the global effect of compensatory changes resulting from the G6PD A- variant as well as alternative endogenous sources of NADPH in nucleated cells. We previously reported that this humanized mouse model demonstrate G6PD activity that is ~ 5% of WT RBCs ^21^. However, G6PD A- bone marrow-derived macrophage NADPH production is ~ 20% reduced compared to WT G6PD activity (Supplementary Fig. 1). It is noteworthy that the inflammatory phenotype in response to bacterial lung infection by these G6PD A- humanized mice is relatively modest, as there were no significant differences in lung vascular permeability or cellular infiltration. In conclusion, this study suggests that G6PD A- variant associated differences in H_2_O_2_ production may drive an enhanced acute immune response to *K. pneumoniae*. Future studies using this humanized mouse model may be useful in defining the impact of G6PD in regulating the ability of non-erythroid cells to handle cellular ROS and their function during states of oxidative stress. Our study directly tests the effect of the X-linked G6PD A- variant on host immune response to *K. pneumoniae*. Because this variant affects approximately 11% of African American men, it may reflect an important, previously unappreciated disease modifier in patients with pneumonia and sepsis.

## Data Availability

The datasets generated during and/or analysed during the current study are available from the corresponding author on reasonable request.

## References

[1] Luzzatto L., Nannelli C. & Notaro R. Glucose-6-Phosphate Dehydrogenase Deficiency. Hematol. Oncol. Clin. North Am. 30, 373–393 (2016). 27040960 10.1016/j.hoc.2015.11.006

[2] Glucose-6-phosphate dehydrogenase deficiency. WHO Working Group. Bull. World Health Organ. 67, 601–611 (1989). 2633878 PMC2491315

[3] McMullin M. F. The molecular basis of disorders of red cell enzymes. J. Clin. Pathol. 52, 241–244 (1999). 10474511 10.1136/jcp.52.4.241 PMC501323

[4] Pamba A. Clinical spectrum and severity of hemolytic anemia in glucose 6-phosphate dehydrogenase-deficient children receiving dapsone. Blood 120, 4123–4133 (2012). 22993389 10.1182/blood-2012-03-416032

[5] Cappellini M. D. & Fiorelli G. Glucose-6-phosphate dehydrogenase deficiency. Lancet 371, 64–74 (2008). 18177777 10.1016/S0140-6736(08)60073-2

[6] Mbanefo E. C. Association of glucose-6-phosphate dehydrogenase deficiency and malaria: a systematic review and meta-analysis. Sci. Rep. 7, 45963 (2017). 28382932 10.1038/srep45963 PMC5382680

[7] Cappadoro M. Early phagocytosis of glucose-6-phosphate dehydrogenase (G6PD)-deficient erythrocytes parasitized by Plasmodium falciparum may explain malaria protection in G6PD deficiency. Blood 92, 2527–2534 (1998). 9746794

[8] Yen W.-C. Impaired inflammasome activation and bacterial clearance in G6PD deficiency due to defective NOX/p38 MAPK/AP-1 redox signaling. Redox Biol. 28, 101363 (2020). 31707353 10.1016/j.redox.2019.101363 PMC6854078

[9] Siler U. Severe glucose-6-phosphate dehydrogenase deficiency leads to susceptibility to infection and absent NETosis. J. Allergy Clin. Immunol. 139, 212–219.e3 (2017). 27458052 10.1016/j.jaci.2016.04.041

[10] Ghergurovich J. M. A small molecule G6PD inhibitor reveals immune dependence on pentose phosphate pathway. Nat. Chem. Biol. 16, 731–739 (2020). 32393898 10.1038/s41589-020-0533-x PMC7311271

[11] Spolarics Z. Increased incidence of sepsis and altered monocyte functions in severely injured type A- glucose-6-phosphate dehydrogenase-deficient African American trauma patients. Crit. Care Med. 29, 728–736 (2001). 11373456 10.1097/00003246-200104000-00005

[12] Vives Corrons J. L. Severe-glucose-6-phosphate dehydrogenase (G6PD) deficiency associated with chronic hemolytic anemia, granulocyte dysfunction, and increased susceptibility to infections: description of a new molecular variant (G6PD Barcelona). Blood 59, 428–434 (1982). 7055648

[13] van Bruggen R. Deletion of leucine 61 in glucose-6-phosphate dehydrogenase leads to chronic nonspherocytic anemia, granulocyte dysfunction, and increased susceptibility to infections. Blood 100, 1026–1030 (2002). 12130518 10.1182/blood.v100.3.1026

[14] Wilmanski J., Villanueva E., Deitch E. A. & Spolarics Z. Glucose-6-phosphate dehydrogenase deficiency and the inflammatory response to endotoxin and polymicrobial sepsis. Crit. Care Med. 35, 510–518 (2007). 17205013 10.1097/01.CCM.0000254337.50361.2E

[15] Pandolfi P. P. Targeted disruption of the housekeeping gene encoding glucose 6-phosphate dehydrogenase (G6PD): G6PD is dispensable for pentose synthesis but essential for defense against oxidative stress. EMBO J. 14, 5209–5215 (1995). 7489710 10.1002/j.1460-2075.1995.tb00205.x PMC394630

[16] McKenna S. M. & Davies K. J. The inhibition of bacterial growth by hypochlorous acid. Possible role in the bactericidal activity of phagocytes. Biochem. J. 254, 685–692 (1988). 2848494 10.1042/bj2540685 PMC1135139

[17] Winterbourn C. C. & Kettle A. J. Redox reactions and microbial killing in the neutrophil phagosome. Antioxid. Redox Signal. 18, 642–660 (2013). 22881869 10.1089/ars.2012.4827

[18] Xiong H. Distinct Contributions of Neutrophils and CCR2+ Monocytes to Pulmonary Clearance of Different Klebsiella pneumoniae Strains. Infect. Immun. 83, 3418–3427 (2015). 26056382 10.1128/IAI.00678-15 PMC4534658

[19] Wang H. One-step generation of mice carrying mutations in multiple genes by CRISPR/Cas-mediated genome engineering. Cell 153, 910–918 (2013). 23643243 10.1016/j.cell.2013.04.025 PMC3969854

[20] Pelletier S., Gingras S. & Green D. R. Mouse genome engineering via CRISPR-Cas9 for study of immune function. Immunity 42, 18–27 (2015). 25607456 10.1016/j.immuni.2015.01.004 PMC4720985

[21] Zuchelkowski B. E. Brief Report: Hydroxychloroquine does not induce hemolytic anemia or organ damage in a “humanized” G6PD A- mouse model. PLoS ONE 15, e0240266 (2020). 33007039 10.1371/journal.pone.0240266 PMC7531777

[22] Zhao Y. Thrombospondin-1 restrains neutrophil granule serine protease function and regulates the innate immune response during Klebsiella pneumoniae infection. Mucosal Immunol. 8, 896–905 (2015). 25492474 10.1038/mi.2014.120 PMC4465063

[23] Lee J. S. The Duffy antigen modifies systemic and local tissue chemokine responses following lipopolysaccharide stimulation. J. Immunol. 177, 8086–8094 (2006). 17114483 10.4049/jimmunol.177.11.8086 PMC2665269

[24] Mangalmurti N. S. Loss of red cell chemokine scavenging promotes transfusion-related lung inflammation. Blood 113, 1158–1166 (2009). 19064726 10.1182/blood-2008-07-166264 PMC2635081

[25] Lee J. S. TLR-4 pathway mediates the inflammatory response but not bacterial elimination in E. coli pneumonia. Am. J. Physiol. Lung Cell. Mol. Physiol. 289, L731–8 (2005). 16024722 10.1152/ajplung.00196.2005

[26] Peñaloza H. F. L-Arginine Enhances Intracellular Killing of Carbapenem-Resistant Klebsiella pneumoniae ST258 by Murine Neutrophils. Front. Cell. Infect. Microbiol. 10, 571771 (2020). 33282749 10.3389/fcimb.2020.571771 PMC7691228

[27] Pineda-Torra I., Gage M., de Juan A. & Pello O. M. Isolation, Culture, and Polarization of Murine Bone Marrow-Derived and Peritoneal Macrophages. Methods Mol. Biol. 1339, 101–109 (2015). 26445783 10.1007/978-1-4939-2929-0_6

[28] DeVallance E. R. Specificity Protein 1-Mediated Promotion of CXCL12 Advances Endothelial Cell Metabolism and Proliferation in Pulmonary Hypertension. Antioxidants (Basel) 12, (2022). 10.3390/antiox12010071 PMC9854820 36670936

[29] de Jesus D. S. Nox1/Ref-1-mediated activation of CREB promotes Gremlin1-driven endothelial cell proliferation and migration. Redox Biol. 22, 101138 (2019). 30802716 10.1016/j.redox.2019.101138 PMC6395885

[30] Li Y. NADPH oxidase 2 inhibitors CPP11G and CPP11H attenuate endothelial cell inflammation & vessel dysfunction and restore mouse hind-limb flow. Redox Biol. 22, 101143 (2019). 30897521 10.1016/j.redox.2019.101143 PMC6435978

[31] Wilmanski J., Siddiqi M., Deitch E. A. & Spolarics Z. Augmented IL-10 production and redox-dependent signaling pathways in glucose-6-phosphate dehydrogenase-deficient mouse peritoneal macrophages. J. Leukoc. Biol. 78, 85–94 (2005). 15817708 10.1189/jlb.0105010

[32] Broug-Holub E. Alveolar macrophages are required for protective pulmonary defenses in murine Klebsiella pneumonia: elimination of alveolar macrophages increases neutrophil recruitment but decreases bacterial clearance and survival. Infect. Immun. 65, 1139–1146 (1997). 9119443 10.1128/iai.65.4.1139-1146.1997 PMC175109

[33] Olonisakin T. F. Stressed erythrophagocytosis induces immunosuppression during sepsis through heme-mediated STAT1 dysregulation. The Journal of Clinical Investigation (2021). 10.1172/JCI137468 PMC7773401 32941182

[34] Nguyen G. T., Green E. R. & Mecsas J. Neutrophils to the roscue: mechanisms of NADPH oxidase activation and bacterial resistance. Front. Cell. Infect. Microbiol. 7, 373 (2017). 28890882 10.3389/fcimb.2017.00373 PMC5574878

[35] Parsanathan R. & Jain S. K. G6PD deficiency shifts polarization of monocytes/macrophages towards a proinflammatory and profibrotic phenotype. Cell. Mol. Immunol. 18, 770–772 (2021). 32523113 10.1038/s41423-020-0428-5 PMC8027810

[36] Imlay J. A. Where in the world do bacteria experience oxidative stress? Environ. Microbiol. 21, 521–530 (2019). 30307099 10.1111/1462-2920.14445 PMC7301649

[37] Lingnau M., Höflich C., Volk H.-D., Sabat R. & Döcke W.-D. Interleukin-10 enhances the CD14-dependent phagocytosis of bacteria and apoptotic cells by human monocytes. Hum. Immunol. 68, 730–738 (2007). 17869646 10.1016/j.humimm.2007.06.004

[38] Peñaloza H. F., Salazar-Echegarai F. J. & Bueno S. M. Interleukin 10 modulation of neutrophil subsets infiltrating lungs during Streptococcus pneumoniae infection. Biochem. Biophys. Rep. 13, 12–16 (2018). 29226257 10.1016/j.bbrep.2017.11.004 PMC5714253

[39] Poe S. L. STAT1-regulated lung MDSC-like cells produce IL-10 and efferocytose apoptotic neutrophils with relevance in resolution of bacterial pneumonia. Mucosal Immunol. 6, 189–199 (2013). 22785228 10.1038/mi.2012.62 PMC3505806

[40] Peñaloza H. F. Interleukin-10 Produced by Myeloid-Derived Suppressor Cells Provides Protection to Carbapenem-Resistant Klebsiella pneumoniae Sequence Type 258 by Enhancing Its Clearance in the Airways. Infect. Immun. 87, (2019). 10.1128/IAI.00665-18 PMC6479034 30804104

[41] Yi H. G6pd-Deficient Mice Are Protected From Experimental Cerebral Malaria and Liver Injury by Suppressing Proinflammatory Response in the Early Stage of Plasmodium berghei Infection. Front. Immunol. 12, 719189 (2021). 34456927 10.3389/fimmu.2021.719189 PMC8386684

